# Supplementary material for: Serum Levels of miR-122-5p and miR-125a-5p Predict Hepatotoxicity Occurrence in Patients Undergoing Autologous Hematopoietic Stem Cell Transplantation
Source: Int J Mol Sci. 2024 Apr 15;25(8):4355. doi: 10.3390/ijms25084355 (PMC11050045; doi:10.3390/ijms25084355)
Supplement: Supplementary file 1 [file ijms-25-04355-s001.zip › Captions of Supplementary Materials.pdf]

**Figure S1.** ROC analysis of analyzed miRNAs for hepatic injury in autologous hematopoietic stem cell transplantation recipients. (**A**) miR-122-5p. (**B**) miR-122-3p. (**C**) miR-99b-5p. (**D**) miR-125a-5p. (**E**) miR-15b-5p.

**Figure S2.** Target prediction analysis of miRNAs included in the model predicting hepatotoxicity occurrence in AHSCT recipients—miR-122-5p and miR-125a-5p. Genes involved in the Hepatitis C KEGG pathway were annotated.

**Figure S3.** Functional enrichment analysis of identified targets of miR-122-5p and miR-125a-5p using the KEGG database.

**Supplementary File S1.** Normalized miRNA expression data with clinical variables.

**Supplementary File S2.** Predicted target genes of miR-122-5p and miR-125a-5p using miRNet and miRTarBase v 8.0.

**Supplementary Table S1.** Multivariate logistic regression analysis for hepatic toxicity (HT) during hospitalization after ASCT—MM patients only.
