# Supplementary material for: Serum Levels of miR-122-5p and miR-125a-5p Predict Hepatotoxicity Occurrence in Patients Undergoing Autologous Hematopoietic Stem Cell Transplantation
Source: Int J Mol Sci. 2024 Apr 15;25(8):4355. doi: 10.3390/ijms25084355 (PMC11050045; doi:10.3390/ijms25084355)
Supplement: Supplementary file 1 [file ijms-25-04355-s001.zip › Supplementary Table S1.pdf]

Supplementary Table S1. Multivariate logistic regression analysis for hepatic toxicity (HT) during hospitalization after ASCT—Results in multiple myeloma patients only.

Variable	Value	OR	95% CI Lower	95% CI Upper	*p*-Value
**Sex**	Male	*Reference*			
	Female	2.29	0.53	10.02	0.2695
**Age at ASCT**	<60 years	*Reference*			
	≥60 years	0.27	0.05	1.32	0.1043
**hsa-miR-122-5p**	1 dCt	2.35	1.28	4.32	0.0058
**hsa-miR-125a-5p**	1 dCt	0.17	0.04	0.82	0.0269
**Conditioning regimen**	Mel-200	*Reference*			
	Mel-reduced	0.52	0.09	2.95	0.4628
