# Supplementary figures and images for: Serum Levels of miR-122-5p and miR-125a-5p Predict Hepatotoxicity Occurrence in Patients Undergoing Autologous Hematopoietic Stem Cell Transplantation
Source: Int J Mol Sci. 2024 Apr 15;25(8):4355. doi: 10.3390/ijms25084355 (PMC11050045; doi:10.3390/ijms25084355)

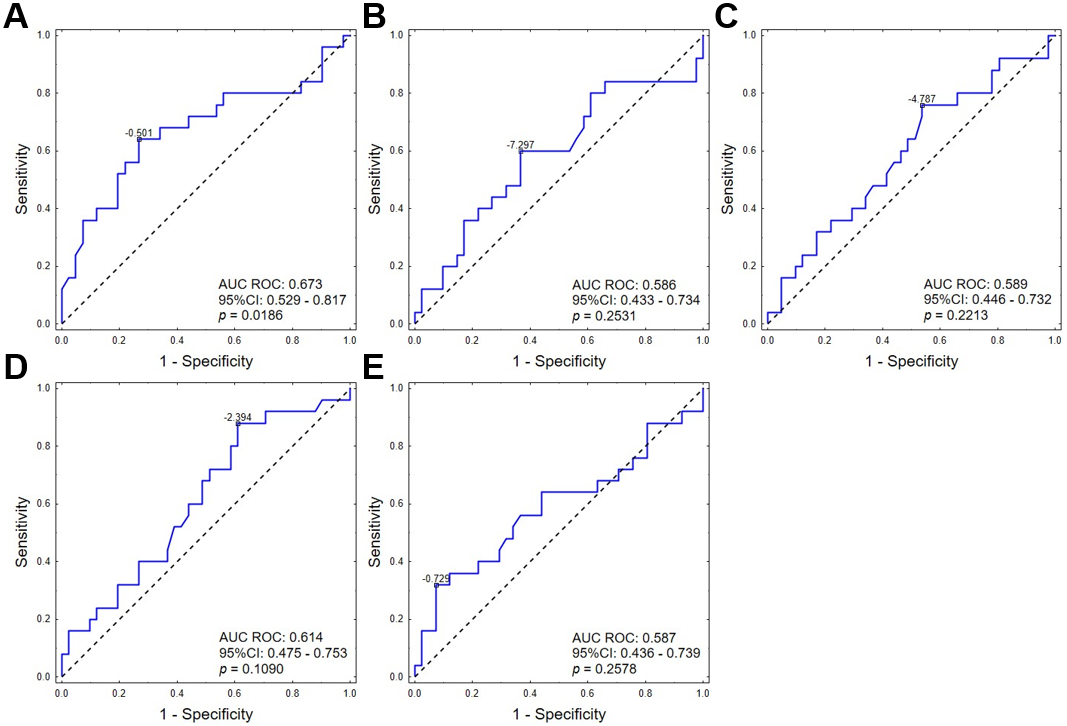

Supplement: Supplementary file 1 [file ijms-25-04355-s001.zip › Supplementary Figure S1.png]

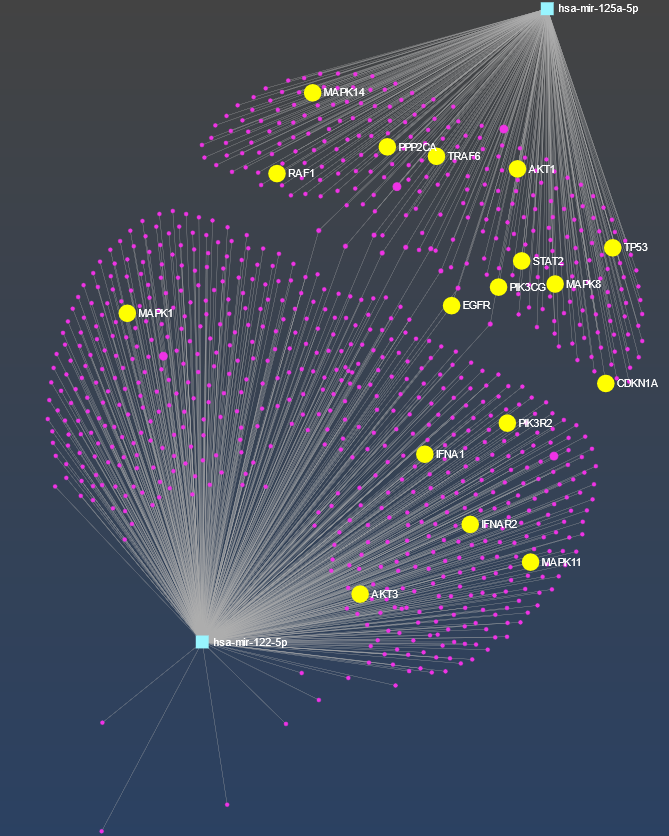

Supplement: Supplementary file 1 [file ijms-25-04355-s001.zip › Supplementary Figure S2.png]

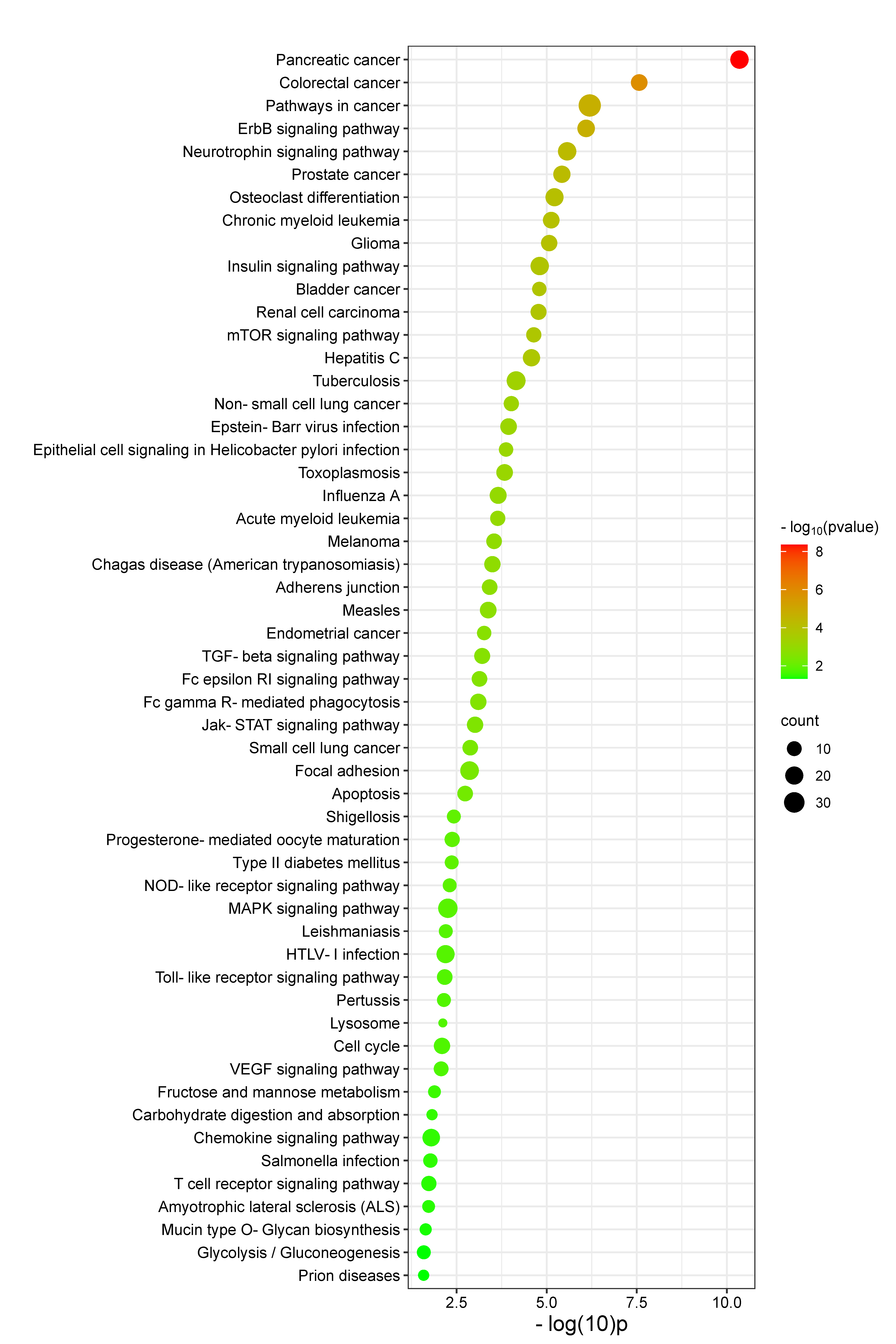

Supplement: Supplementary file 1 [file ijms-25-04355-s001.zip › Supplementary Figure S3.tiff]
